# Experimental study on shear mechanical behavior of bolted rock joints and rock-bolt interaction mechanism

**DOI:** 10.1038/s41598-025-34502-5

**Published:** 2026-01-06

**Authors:** Shan Deng, Zihao Sun, Linfeng Zhu, Luobin Zheng, Meng Lv, Xuchen Wang, Chenlu Wang, Zhongjun Ma

**Affiliations:** 1 Faculty of Engineering, China University of Geosciences, Wuhan, 430074 China; 2 School of Resources and Environmental Engineering, Wuhan University of Science and Technology, Wuhan, 430081 China; 3 Institute of Rock Mechanics, Ningbo University, Ningbo, 315211 China

**Keywords:** Bolted joints, Shear mechanical behavior, Mechanical interaction mechanism, Axial force monitoring, JRC, Engineering, Materials science

## Abstract

Rock bolting controls shear slip along rock joints, yet the deformation coordination and mechanical response during shearing are not well understood due to complex bolt-rock interaction. This study systematically conducted direct shear tests to investigate how the joint roughness coefficient (JRC), normal stress, and bolt inclination affect the shear behavior of bolted joints. A custom-designed monitoring system was employed to continuously measure the bolt axial force throughout the shearing process. Results indicate that JRC strongly influences bolt deformation and axial-force mobilization through dilatancy, thereby governing the peak and reinforced strengths of the joint. Higher normal stress suppresses joint dilatancy and reduces the bolt’s shear contribution, while bolt inclination controls the balance between axial and shear forces. 45° inclination showed the best overall shear performance. Monitoring data reveal the bolt’s axial force variation during shear displacement progresses through four stages: initial adjustment–relaxation, elastoplastic, hardening, and deformation-failure. This classification tracks how axial force evolves under compatible coordination. Correspondingly, the bolted joint’s shear process develops through elastic, plastic, failure, and residual stages, revealing the mechanical interaction mechanism between the joint surface and bolt at different stages. The study provides an experimental basis for understanding shear behavior of bolted joints and optimizing rock bolt support design.

## Introduction

Rock bolting technology, due to its cost-effectiveness and adaptability, has been extensively applied in various geotechnical engineering projects^1–3^. Historically, the transverse shear resistance of rock bolts was often neglected, as bolts were typically idealized as pure tension members designed to stabilize potentially unstable rock blocks. Consequently, early research primarily focused on their load-transfer behavior and failure modes under axial load control^4–7^. In complex geological environments where rock masses contain discontinuities of varying scales^8–11^, bolts that cross these structural planes and anchor into stable bedrock can effectively restrict interlayer slippage, enhance joint shear resistance, and thereby improve the overall stability of rock masses. However, bolts near the joint surface are simultaneously subjected to tensile and shear stresses, resulting in coupled deformation and potential failure^12,13^. Therefore, understanding the mechanical interaction and deformation coordination between the bolt and joint is crucial for improving reinforcement design.

The shear strength of a bolted rock joint can be generally considered to consist of two parts: the inherent shear resistance of the joint itself and the additional shear contribution provided by the bolt^14–16^. While the shear strength of untreated rock joints has been extensively studied, research on the shear contribution of bolts within bolted joints still lags behind engineering practice. Experimental investigations under various influencing factors have been conducted to explore the shear behavior of bolted joints, leading to several valuable insights.

For jointed rock masses, both rock strength and joint surface characteristics play critical roles in governing the mechanical behavior of bolted rock joints. Laboratory shear tests on bolted joints using rocks or concrete materials of different strengths have demonstrated that higher rock strength enhances the transverse shear effect of bolts, thereby increasing their contribution to joint shear strength^14,17,18^. Ferrero^19^ reported that the shear performance of bolts is strongly correlated with rock strength: when the uniaxial compressive strength exceeds 50 MPa, bolts tend to fail in a tensile–shear mode, whereas at lower strengths, tensile–bending failure is more likely to occur. The morphology of the joint surface directly affects the dilatancy angle of the joint, alters the normal displacement, and consequently influences bolt stress, deformation, and the overall shear behavior of bolted rock joints. Spang and Egger^14^ observed significant differences in bolt shear contribution between rough and smooth joint surfaces. Yoshinaka et al.^20^ showed that, for bolted joints with sawtooth-shaped surfaces, a stronger dilatancy effect results in more pronounced axial-force mobilization in the bolt. Wu et al.^21^ confirmed through model tests that increased roughness enhances both normal displacement and bolt shear resistance. Chen et al.^22^ also found that increasing normal stress reduces bolt deformation, while higher roughness enhances it. Wang et al.^23^ concluded that both peak and residual shear strengths increased with joint surface roughness.

Other researchers have conducted experimental studies on the shear performance of bolted joints focusing on various bolting parameters. Bolt inclination angle plays a vital role in determining the interaction between shear and axial forces within the bolt. A steeper inclination amplifies the dowel action and increases the shear component, while a shallower angle promotes axial tension. The optimal inclination angle for bolted joints typically ranges between 30° and 60°^24–26^. Other parameters, such as pre-stress^27^, anchorage length^28^, bolt material and type^29–31^, diameter^32^, number of bolts^33^, and grout material^34^.

Despite considerable progress, existing research still faces several limitations. Previous studies have mainly analyzed the evolution of shear strength and anchoring mechanisms under one or more influencing factors. However, since bolts are embedded within rock masses and surrounded by grout, it is often difficult to directly monitor the complete stress evolution and failure process of the bolt during shear. Consequently, most earlier research focused on the overall shear mechanical response of bolted joints, without clarifying the deformation coordination between the joint and the bolt or the detailed mechanical response of the bolt throughout the shear process. Moreover, bolt deformation during shear is strongly influenced by the three-dimensional morphology of the joint surface, which in turn affects the mechanical behavior of the bolt. Previous studies often employed simplified saw-tooth or quasi-3D joint geometries, which cannot accurately reproduce the shear mechanical behavior and deformation coordination of natural irregular joint surfaces.

Therefore, this study employs red sandstone specimens with accurately machined three-dimensional joint morphologies. Direct shear tests were conducted under varying joint roughness, normal stress, and bolt inclination angles. The bolt axial force was continuously monitored using an embedded axial force sensor, allowing real-time observation of deformation and failure evolution. Based on the variations in shear load, joint dilatancy, and bolt axial force, the shear process was divided into distinct mechanical stages, and the deformation coordination and anchoring mechanisms were systematically analyzed.

## Method

### Testing method and configuration

**Fig. 1:**
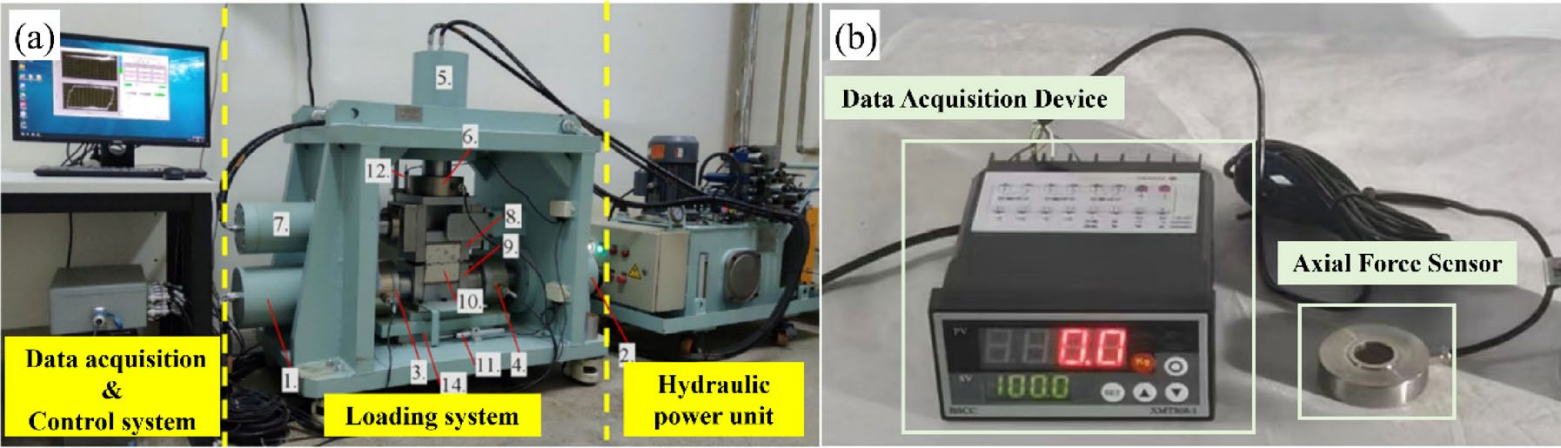
**a** Multi-function rock mass discontinuity shear apparatus; **b** Axial force monitoring system.

The shear testing apparatus employed in this study is the HDL-XHJQ-KJ multifunctional cyclic shear instrument for rock mass structural planes, independently developed by the Institute of Rock and Soil Mechanics, Chinese Academy of Sciences^35^. As shown in Fig. 1a, the instrument comprises three main components: the loading system, the hydraulic power unit, and the data acquisition and control unit.

**Fig. 2:**
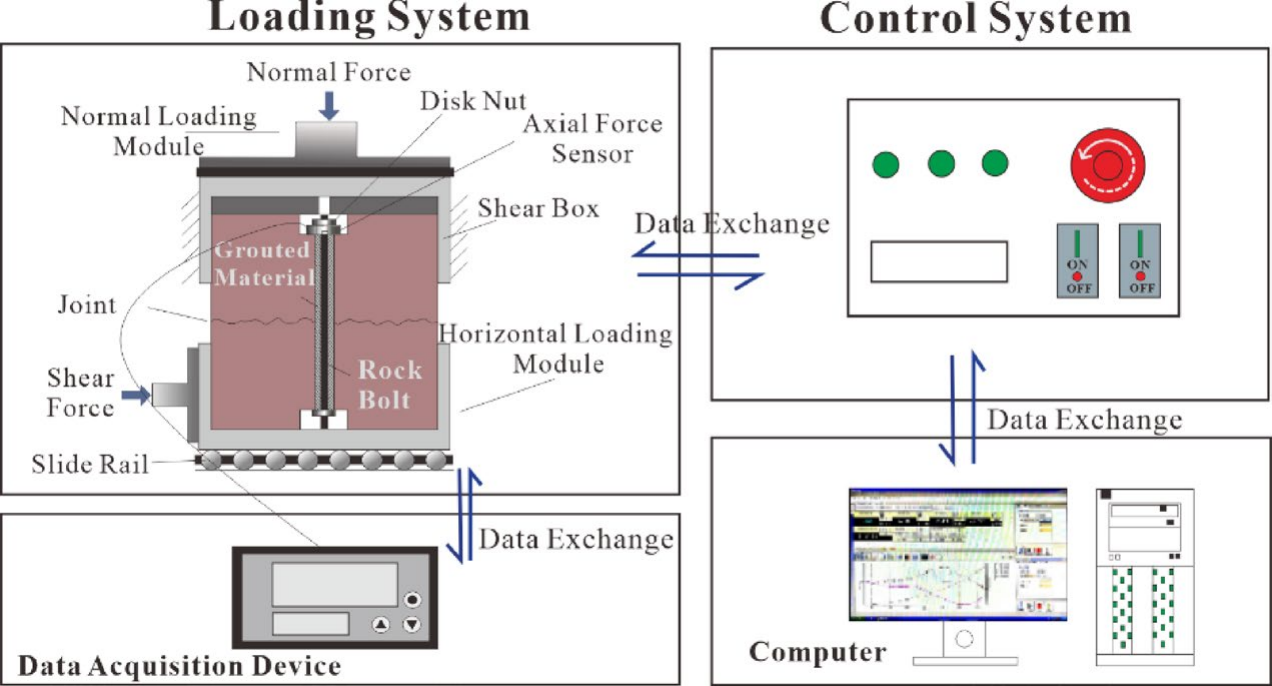
Shear test model of bolted rock joints.

A miniature axial force monitoring device was installed at the anchored end of the rock bolt within the joint, connected to the data acquisition device via data cables to enable real-time measurement of bolt axial force during shear testing. The bolt axial force monitoring system is illustrated in Fig. 1b, and the shear test model is shown in Fig. 2.

The axial force monitoring system was custom-designed by Stailnge Intelligent Technology Co., Ltd. The axial force sensor has a disk diameter of 24 mm, a height of 6.5 mm, and a measurement range of 1.5 tons. The data acquisition unit is connected to both the power supply and the sensor, allowing real-time data collection and on-screen display, with recorded data retrieved via the associated “DataManagement” software (Version 1.0; http://www.stsensor.com). The data acquisition interval can be adjusted; in this experiment, it was set to 1 s.

### Specimen preparation

Sandstone from the Feixianguan Formation (T₁f) of the Lower Triassic strata in southwestern China was selected as the parent rock material. Each specimen was cut into rectangular blocks with dimensions of 150 mm × 150 mm × 77 mm. The specimen preparation process included: (1) drilling a central borehole of 10 mm diameter for bolt insertion; (2) milling a 25 mm diameter, 15 mm deep recess at the upper block center to accommodate the load cell and locking nut; (3) carving a groove (5 mm wide, 15 mm deep) to house the sensor cable; and (4) machining a 25 mm diameter, 10 mm deep recess at the corresponding lower block position to accommodate the lower locking nut.

The three-dimensional surface morphologies of the joint specimens were sculpted using computer numerical control (CNC) carving^36^ based on digital roughness data generated via SynFrac software (Version 1.0; https://homepages.see.leeds.ac.uk/~earpwjg/PG_EN/Software.htm). Four representative joint morphologies with distinct roughness coefficients were selected. The joint roughness coefficient (JRC) was calculated using the following relation.

1 $${Z_2}={\left[ {\frac{1}{{N - 1}}{{\sum\limits_{{i=1}}^{{i=N - 1}} {\left( {\frac{{{y_{i+1}} - {y_i}}}{{{x_{i+1}} - {x_i}}}} \right)} }^2}} \right]^{\frac{1}{2}}}$$

Where *N* is the number of discrete points along the profile, *x*_*i*​_, *y*_*i*​_: the *x*- and *y*-coordinates of the *i*^*th*^ discrete point, respectively. With a sampling interval of 0.5 mm and a joint surface side length of 150 mm, the total number of points is *N* = 301.

Tatone and Grasselli^37^ proposed a regression equation between the joint roughness coefficient (JRC) and *Z₂* for a sampling interval of 0.5 mm:

2 $$JR{C_i}=51.85{\left( {{Z_{2i}}} \right)^{0.6}} - 10.37$$

Where *JRC*_*i*_ is the roughness coefficient of the *i*^*th*^ profile, and *Z*_*2i*_ is the root mean square of the slope corresponding to that profile. It should be noted that the JRC value obtained here is a two-dimensional (2D) value.

The two-dimensional JRC values were extracted from each profile line at the specified sampling interval, and their average was taken as the three-dimensional JRC of the joint surface:

3 $$JR{C_{3D}}=\frac{1}{N}\sum\limits_{{i=1}}^{N} {JR{C_i}}$$

Therefore, the JRC values referred to in the subsequent sections represent the three-dimensional value, *JRC*_*3D*_, and are used as the primary scalar index for quantifying joint roughness in this parametric study.

**Fig. 3:**
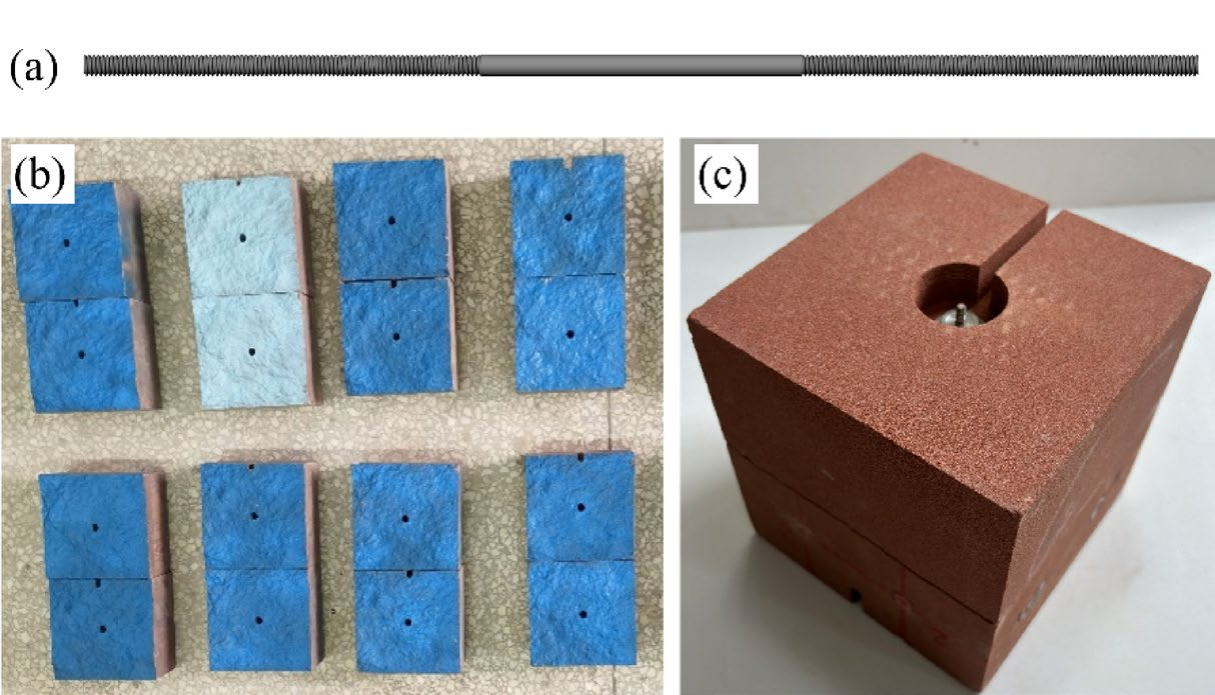
**a** Schematic of the rock bolt structure; **b** Drilling and carving of the intact rock block; **c** Bolted joint specimen.

Following this preparation, the matching upper and lower rock blocks were assembled to form a through-going hard joint surface sample, ready for the subsequent rock bolting procedure. The rock bolts were manufactured from A3 carbon steel, featuring a diameter of 4 mm and threads rolled onto both ends, as illustrated in Fig. 3a.

After tightly bonding the upper and lower blocks (Fig. 3b) using steel straps to form an integrated specimen, the rock bolt was vertically installed into the central borehole of the rock block. The bottom was secured with a washer and disc nut, providing a sealed space for grouting. Grout was injected gradually from the bottom of the borehole upward via a syringe connected to a delivery tube. Upon completion of grouting, the upper locking nut was installed, as shown in Fig. 3c, and the specimen was left to rest for 24 h before being cured in a conditioning chamber for 28 days.

Material properties of the rock, grout, and bolt are summarized in Tables 1 and 2.

**Table 1:** Parameter values of rock and grout.

Property	Density/(g/cm^3^)	Elastic modulus /GPa	Poisson’s ratio	Uniaxial compressive strength /MPa	Joint friction angle /°	Cohesion /MPa
Rock	2.39	13.43	0.27	51.44	35	6.26
Grout	2.04	10.66	0.18	29.23	–	–

**Table 2:** Parameter values of the rock bolt.

Density/(g/cm^3^)	Elastic modulus /GPa	Poisson’s ratio	Yield strength /MPa	Ultimate tensile strength/MPa	Elongation/%
7.85	200	0.25	420	615	13.5

### Test procedure

The axial force sensor was installed in the reserved borehole of the upper rock block, and the specimen was placed into the shear box after alignment of the upper and lower shear boxes. Subsequently, the normal displacement was increased at a rate of 1 mm/min to bring the top and bottom walls closer together. The normal load was applied using hydraulic oil via a servo-controlled hydraulic pump at a rate of 0.5 kN/s until the target normal stress was reached. Once the normal stress was applied, the upper plate was fixed, and the shearing load was applied to the lower plate, maintaining a horizontal shear rate of 1 mm/min. Loading was stopped when the bolt fractured and shearing continued for an additional 2 mm, or when the shear displacement reached 15 mm. Throughout the shearing process, the normal stress was servo-controlled with an accuracy of ± 0.001 MPa, and the shear displacement automatically reversed direction when the displacement amplitude limit was reached. Normal displacement was measured by a linear variable differential transducer (LVDT) with a range of 15 mm, while the relative horizontal displacement of the two parts of the fracture specimen was measured by an LVDT with a 50 mm range. Axial force data were recorded every second by the data acquisition system.

Direct shear tests under a normal stress of 1 MPa were conducted on bolted rock joints with varying roughness coefficients (JRC = 3.14, 7.99, 12.01, 16.07, or smooth joint).

For the rough bolted rock joints with JRC = 12.01, a series of direct shear tests were performed under different normal stress levels (*σ*_n_ = 0.5 MPa, 1 MPa, 2 MPa). The normal stress levels (0.5, 1, and 2 MPa) were selected to simulate in-situ conditions in common rock engineering structures reinforced by bolts, such as slopes and shallow tunnels. This range corresponds to overburden depths of approximately 20–80 m, representing a typical operational range for systematic rock bolting.

For bolted smooth joints, direct shear tests were conducted at different bolt inclinations (30°, 45°, 60°, 90°).

Each unique test condition was conducted once, with the high consistency from CNC machining, strict protocol standardization, and clear cross-parameter trends collectively affirming the reliability of the results. These experiments were designed to systematically investigate the influence of these key parameters on the shear mechanical behavior of bolted rock joints.

## Results

### Shear behavior with different roughness coefficients

Direct shear tests were conducted on bolted rock joints with five different roughness coefficients (JRC = 0, 3.14, 7.99, 12.01, 16.07), and the results are presented as follows.

**Fig. 4:**
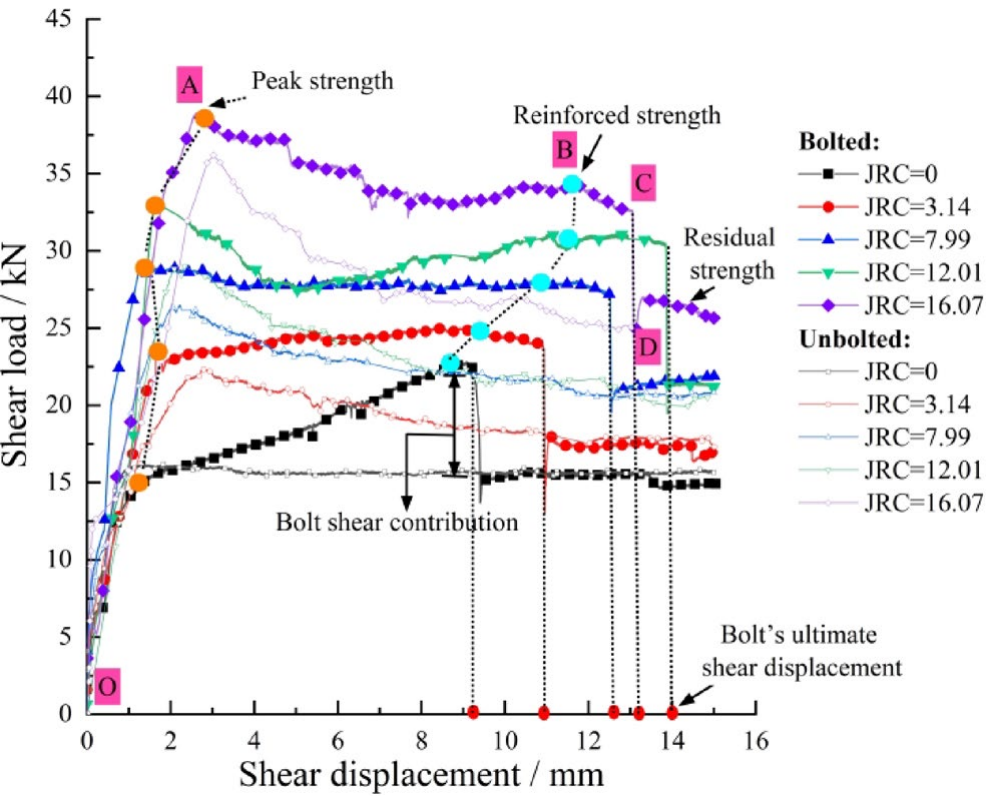
Shear load–shear displacement curves of bolted/unbolted joints with different JRC.

**Fig. 5:**
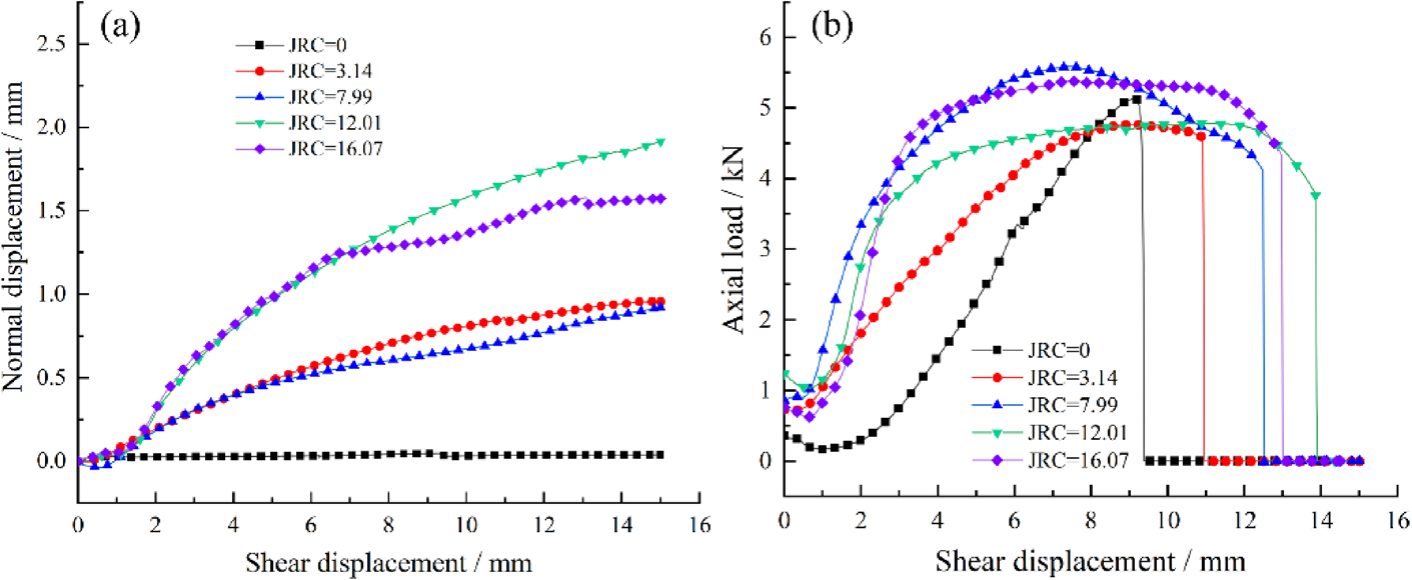
**a** Dilatancy curves of bolted joints with different JRC; **b** Axial force–shear displacement curves of bolted joints with different JRC.

As illustrated in Fig. 4, shear load evolution differs significantly between bolted and unbolted joints. Analysis shows the joint itself dominates initial shear strength, while bolts only marginally improve shear stiffness. After the peak shear strength of the joint is attained, the bolt progressively mobilizes its shear resistance with increasing displacement due to deformation coordination. At this stage, the overall shear response becomes a combined function of the inherent shear strength of the joint surface and the shear contribution of the bolts.

During the initial stage of shearing (OA), the shear response of the bolted rock joint is primarily controlled by the joint surface itself, exhibiting high shear stiffness. The first inflection point on the shear load–displacement curve corresponds to the peak strength of the bolted joint. With further increase in shear displacement (AB), the bolt enters a plastic hardening stage, during which it provides additional shear resistance to the joint. The maximum value of this enhanced resistance is defined as the reinforced strength of the bolted joint, representing the peak shear capacity contributed by the bolt through its axial and shear forces after the inherent strength of the joint itself has been mobilized and begun to degrade. As shearing continues and the bolt approaches its ultimate capacity (BCD), necking develops, leading to a gradual reduction in load-bearing capacity, followed by fracture, which manifests as a sharp drop on the shear load curve. The shear displacement at the point of fracture is defined as the ultimate shear displacement of the bolt. Beyond this point, the curve levels off, signifying the residual strength stage of the joint.

With increasing JRC values, the contribution of the bolt to the peak shear strength of the joint during the initial shearing stage becomes increasingly pronounced. Analysis of the normal displacement of the bolted joints in Fig. 5a further reveals that under identical shear displacements, a higher JRC induces greater normal displacement, leading to more significant bolt deformation and thereby generating higher axial force.

Figures 4 and 5b show that bolt shear resistance between 6 and 10 kN and bolt-end peak axial force of 4.5–5.5 kN. Higher JRC generally increases shear displacement to reach reinforced and ultimate strength, as greater normal displacement provides more axial deformation space, effectively mobilizing the bolt’s dowel capacity. However, at JRC = 16.07, ultimate shear displacement decreases, likely from excessive normal displacement causing premature failure.

### Shear behavior under varying normal stress

For the rough bolted rock joints with JRC = 12.01, a series of direct shear tests were performed under different normal stress levels (*σ*_n_ = 0.5 MPa, 1 MPa, 2 MPa).

**Fig. 6:**
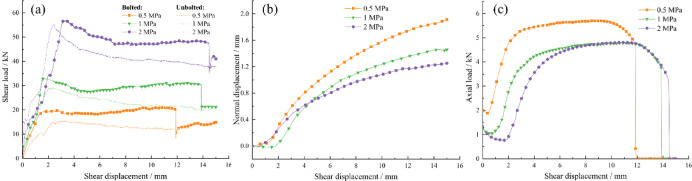
Shear behavior curves of bolted joints under different normal stresses. **a** Shear load–shear displacement curves under different normal stresses of bolted and unbolted joints; **b** Dilatancy curves under different normal stresses; **c** Axial force–shear displacement curves under different normal stresses.

Figure 6a shows that the bolt significantly enhances joint shear stiffness during the initial shearing stage under 0.5 MPa normal stress, whereas this effect weakens as normal stress increases to 1–2 MPa.

Figure 6b shows that the higher normal stress suppresses joint normal displacement, thereby limiting bolt deformation and reducing its mobilized shear resistance under the same shear displacement.

Figure 6c shows that lower normal stress promotes earlier mobilization of bolt shear resistance and leads to smaller ultimate shear displacement at fracture, due to rapid mobilization of the bolt’s shear capacity accelerating its failure.

Moreover, the consistent difference between reinforced and residual strengths across normal stress levels indicates that the bolt’s shear contribution remains essentially stable, which aligns with findings from Haas^38^. This observed insensitivity can be attributed to the primary mechanism of resistance: the bolt’s shear contribution is partly derived from its axial force, which is mobilized by joint dilatancy. While higher normal stress suppresses dilatancy and slows the rate of axial force mobilization, the bolt within this stress range is still able to yield and approach its ultimate tensile capacity, leading to a relatively stable maximum shear contribution.

### Shear behavior with different bolt inclination angles

For the smooth bolted rock joints, Direct shear tests were conducted on unbolted joints and bolted rock joints with bolt inclination angles of 30°, 45°, 60°, and 90°.

**Fig. 7:**
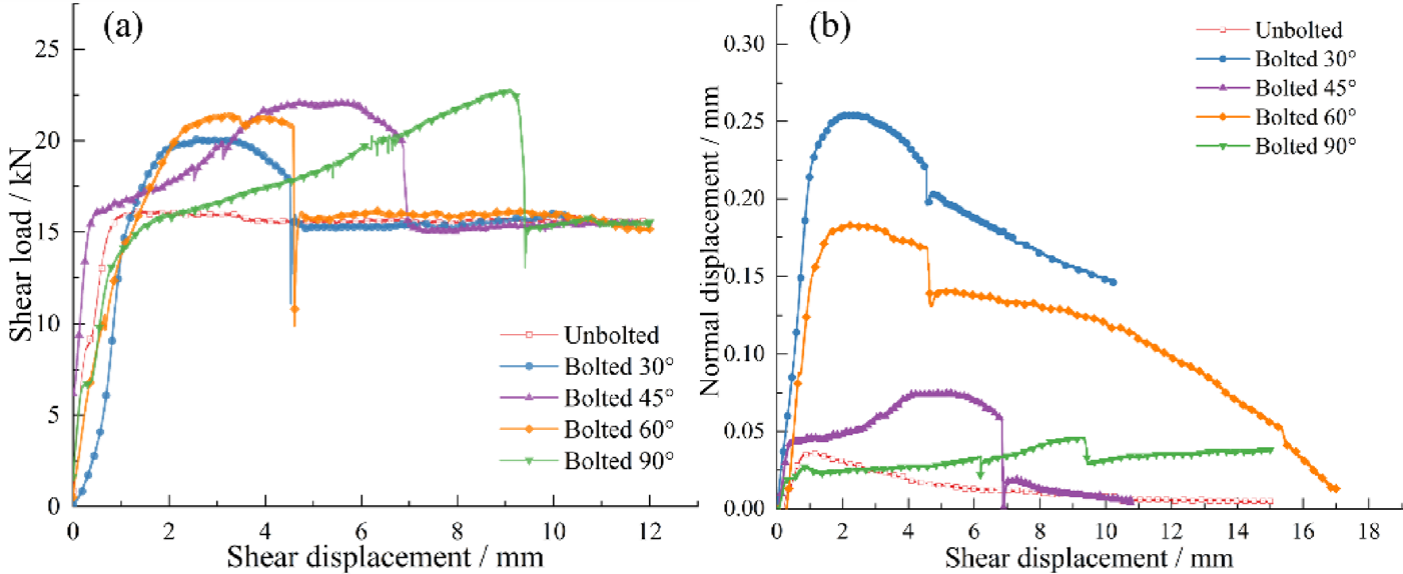
**a** Shear load–shear displacement with different bolt inclination angles; **b** Dilatancy curves of bolted joints with different bolt inclination angles.

Figure 7a shows that the shear strength of bolted joints varies with bolt inclination angle, with peak shear loads of 20.04 kN, 21.40 kN, 22.14 kN, and 22.65 kN for inclinations of 30°, 45°, 60°, and 90°, respectively.

Figure 7b shows that a guiding effect induced by the bolt results in significant differences in the normal displacements across different inclination angles. This “guiding effect” is a result of the kinematic constraint imposed by the inclined bolt. As the joint undergoes shear displacement, the relative movement of the upper and lower blocks is forced to follow a path that is compatible with the fixed length and orientation of the bolt across the joint. For a bolt inclined at 90° (perpendicular to the joint), this constraint is minimal, as shear displacement does not directly stretch the bolt. However, for any other inclination (e.g., 30°, 45°, 60°), shear displacement geometrically forces the two blocks to separate or converge, thereby guiding the normal displacement. The bolt’s axial stiffness then resists this induced normal displacement, generating the axial force that contributes to the shear resistance. Consequently, peak normal displacements of 0.26 mm, 0.08 mm, 0.18 mm, and 0.05 mm were observed for inclinations of 30°, 45°, 60°, and 90°, respectively.

At 30° and 60° bolt inclinations, the bolted joints exhibited higher peak strength but lower reinforced strength and reduced ultimate shear displacement. Under these angles, the bolts underwent significant tensile deformation, mobilizing substantial axial force rapidly at small shear displacements and reaching ultimate strength earlier. This resulted in increased joint peak strength and decreased shear displacement. However, the limited shear displacement restricted full development of transverse shear resistance, leading to lower reinforced strength.

At 90° inclination (perpendicular to the joint), the guiding effect was negligible with minimal normal displacement. Consequently, shear resistance mobilized slowly, but full dowel action yielded the highest reinforced strength. The 45° inclination provided an optimal balance, enabling rapid shear resistance mobilization while maintaining partial dowel action. Thus, 45° was identified as the optimal inclination angle under the tested conditions.

## Discussion

### Bolt mechanical response under coordinated deformation

**Fig. 8:**
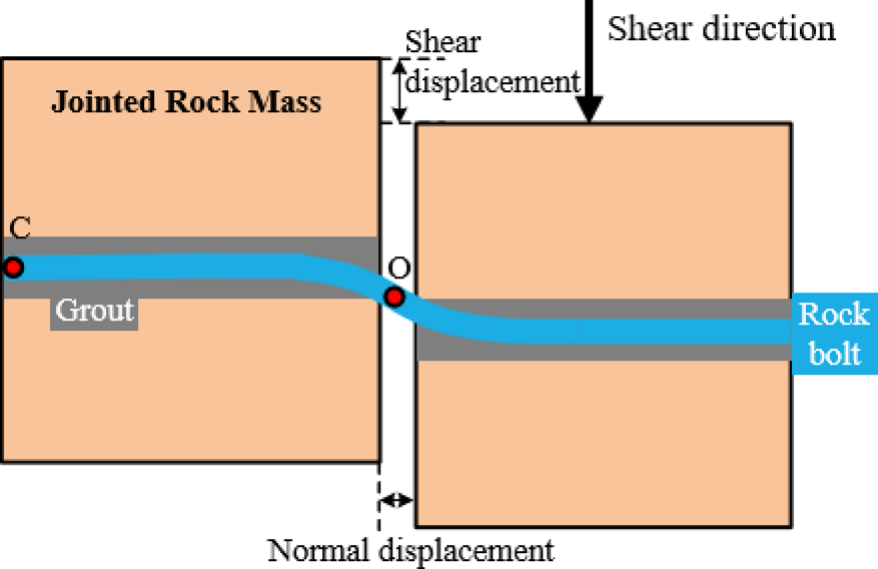
Schematic diagram of bolt deformation during shearing.

The rock bolt, particularly near the joint surface, is subjected to both tensile and shear forces, and its stress state evolves with increasing shear displacement, as illustrated in Fig. 8. According to the dilation curve in Fig. 5a, the normal displacement exhibits a nonlinear positive correlation with shear displacement during the shearing process, indicating that the stress state of the bolt varies at different shear displacements. In this test, the monitored axial force represents the normal force acting on the end of the bolt.

By comparing the shear displacement curve (Fig. 4) with the axial force–displacement curve (Fig. 5b), it can be observed that the latter reflects, to some extent, the shear resistance mobilized by the bolt at various shear displacements. Therefore, based on the mechanical behavior of materials under tension and shear, the axial force–shear displacement curve of the bolt is analyzed to interpret its mechanical response during shearing.

**Fig. 9:**
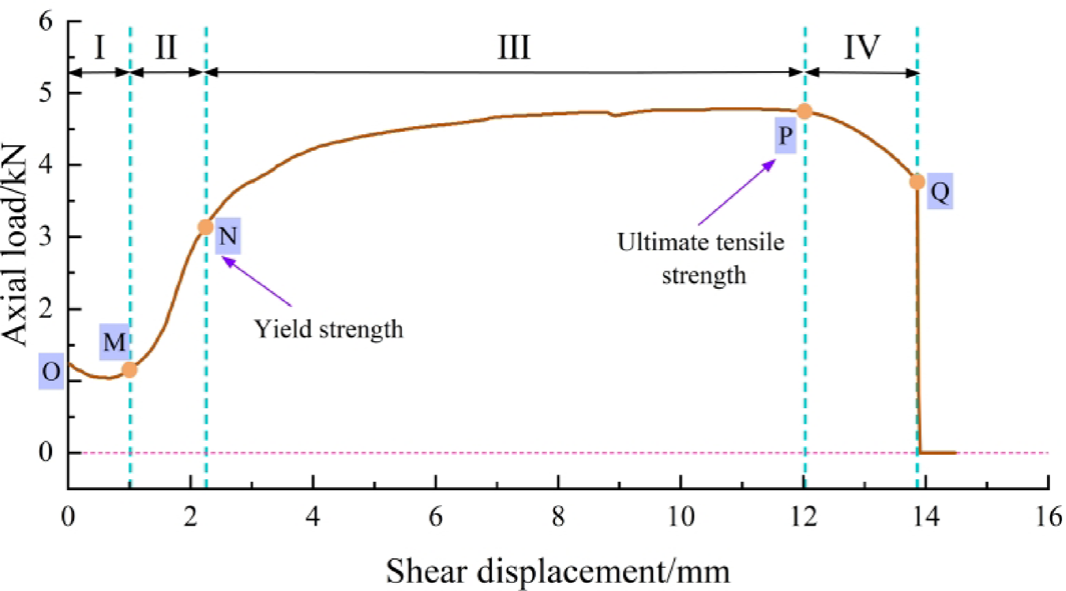
Axial force–shear displacement curve of bolted joints.

Based on the mechanical properties of metal bars and the shearing process, the variation of bolt axial force with shear displacement can be divided into four characteristic stages, as illustrated in Fig. 9.

Stage Ⅰ: Initial adjustment - relaxation stage (OM).

At the onset of shearing (point O), the axial force equals the initial pre-stress value, or zero if no pre-stress is applied. As shear displacement increases, the axial force exhibits a slight decrease or remains essentially unchanged. Point M marks the end of this stage. Its position generally coincides with the end of normal contraction (dilatancy-negative) of the joint. If the joint does not exhibit significant contraction, point M is identified as the location where the axial force, after a brief adjustment, returns to its initial value. This behavior results primarily from two mechanisms:

(i) Interfacial adjustment. Adhesive and frictional interactions at the bolt–grout and grout–rock interfaces undergo micro-slippage and readjustment at the onset of shearing. This partial debonding and frictional loss cause a relaxation of the axial force at the bolt end.
(ii) Initial normal contraction. Surface asperities interlock under early shear and normal loading, generating localized cracking and energy dissipation. Since static friction exceeds kinetic friction, the initiation of slip may induce a short-lived normal contraction (negative dilation), which redistributes stresses along the bolt.

The combined effects of interfacial adjustment and normal contraction yield an initial adjustment–relaxation phase. For smooth joints, the contraction effect is negligible, and the behavior is dominated by interfacial adjustment, often resulting in a prolonged relaxation phase (Fig. 5b). For rough joints, coupled tensile–shear deformation modifies adhesion and friction, resulting in stress relaxation.

Stage Ⅱ: Elastoplastic stage (MN).

In this stage, the bolt axial force increases with shear displacement, driven primarily by joint dilatancy. The associated increase in normal displacement induces bolt elongation, causing the bolt to deform elastically before rapidly yielding and entering the plastic regime. The shape of the curve in this phase is strongly influenced by the magnitude of dilatancy. Under conditions of suppressed dilatancy (e.g., high normal stress or low JRC), the gradual increase in bolt elongation often leads to a prolonged, near-linear rise in axial force, indicative of a dominant elastic response over a large shear displacement. In contrast, under conditions of pronounced dilatancy (e.g., low normal stress or high JRC), the rapid stretching of the bolt typically results in a steeper, more nonlinear increase in axial force and a shorter elastic phase, accelerating the onset of yielding. Point N marks a distinct change in the slope of the axial force curve, where the rate of increase slows. The axial force at this point corresponds to the yield strength of the bolt material, signifying the transition from the elastoplastic stage to the hardening stage.

Stage Ⅲ: Hardening stage (NP).

At this stage, the axial force continues to increase, but at a reduced rate. This phenomenon is associated with the combined effect of diminished joint dilatancy and the onset of strain hardening in the bolt. As shearing progresses, asperities are sheared off, and the dilation angle decreases, slowing the rate of normal displacement increase. Concurrently, the bolt material enters its strain-hardening regime, where the rate of axial-force growth with strain decreases. These effects jointly result in a gentler slope until point P, where the axial force reaches its ultimate strength.

Stage Ⅳ: Deformation - fracture stage (PQ).

In the final stage, the axial force decreases nonlinearly until the bolt fractures at point Q. Continued shear and dilation lead to localized plastic deformation and necking, reducing the bolt’s cross-sectional area and load-bearing capacity. Consequently, the axial force–shear displacement curve descends until the bolt completely ruptures, and the axial force drops rapidly to zero.

In summary, the four stages of bolt axial force evolution provide a data-driven framework to deconstruct the bolt’s mechanical response during shearing. This bolt-centric perspective is key to understanding the deformation coordination mechanism and the resulting macroscopic shear behavior of the bolted joint system, which is analyzed in detail in the next section.

### Mechanical interaction in the rock-bolt system

**Fig. 10:**
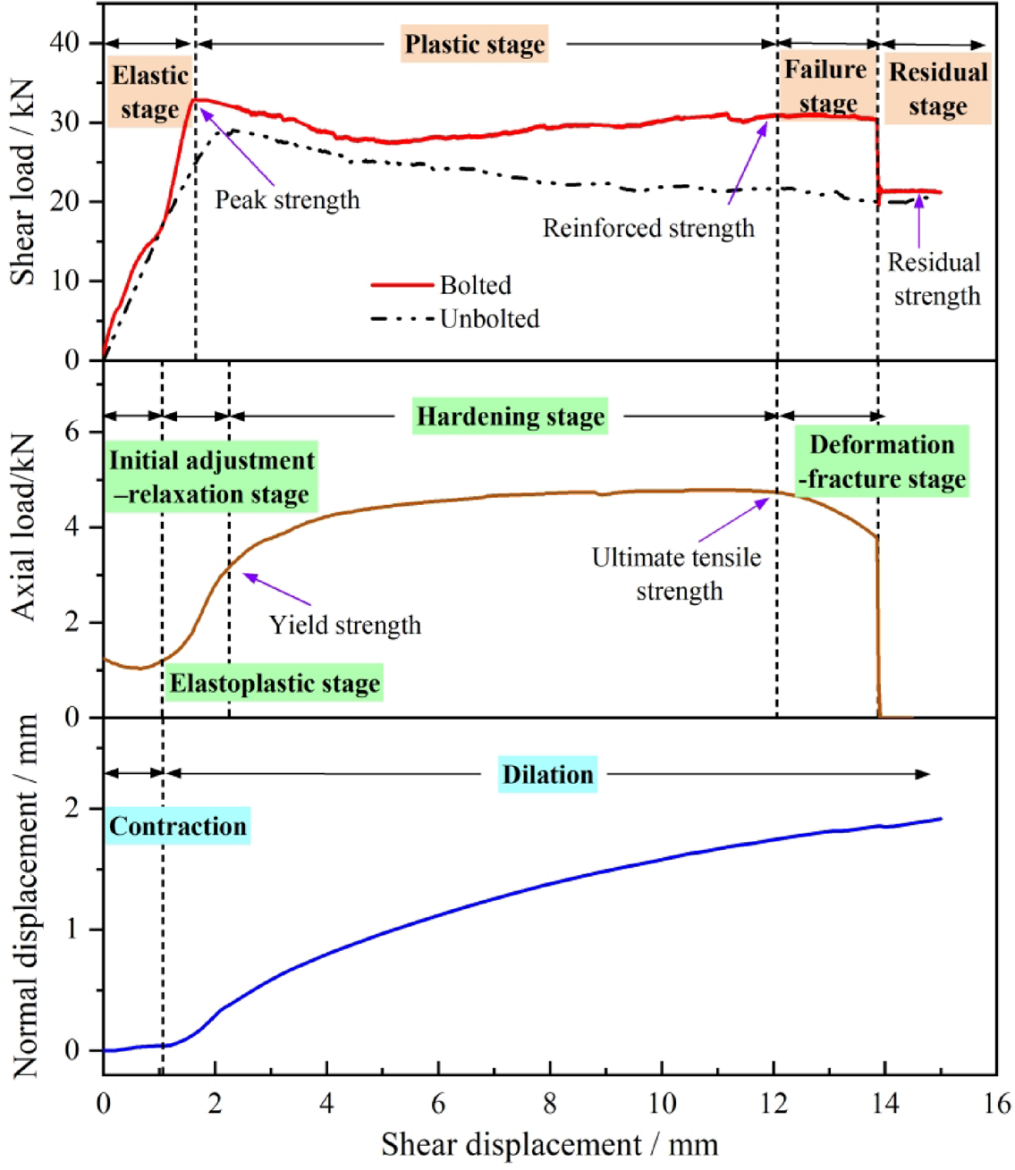
Characteristic stages of the shear mechanical behavior for bolted joints.

By synthesizing the variations in shear load, dilation, and bolt axial force with shear displacement, the shearing process can be divided into four stages: the elastic stage, plastic stage, failure stage, and residual stage, shown in Fig. 10. The shear load–displacement curves of joints with JRC = 12.01 under a normal stress of 1 MPa and a bolt inclination of 90° were aligned along a common shear displacement axis for detailed interpretation.

(1) Elastic stage. In this stage, the shear load increases nearly linearly and reaches its peak at a shear displacement of 1–3 mm. The response of bolted joints resembles that of unbolted joints but with a steeper slope and higher strength, reflecting the combined contribution of the joint surface and the bolt. Because normal displacement remains small, the bolt’s axial deformation is limited, and its axial force lies within the adjustment–relaxation or early elastoplastic phases. The increase in shear resistance compared with unbolted joints is therefore primarily due to the bolt’s transverse (shear) resistance, supplemented by the normal component of the bolt axial force (or prestress) along the joint plane.

(2) Plastic stage. The shear load–displacement response during this stage exhibits two distinct patterns, plastic hardening and plastic softening, which are governed by the competing mechanisms of joint degradation and bolt reinforcement.

For context, unbolted joints typically demonstrate either brittle failure, characterized by sharp post-peak stress drops, or ductile behavior, exhibiting gradual strength reduction or nearly constant residual strength^39^. For bolted joints, the post-peak behavior is modified through the bolt’s interaction with the joint. The bolt mobilizes resistance through coordinated deformation, a process in which joint dilatancy plays a decisive role. This dilatancy, which is enhanced by surface roughness yet suppressed by normal stress, induces greater axial deformation in the bolt. Consequently, the bolt’s contribution to the overall shear resistance is amplified via the generation of axial force.

The post-peak response of a bolted joint is a competition between the strength degradation of the joint itself and the mobilization of the bolt’s resistance. The phenomenon of plastic hardening or plastic softening emerges from the outcome of this competition.

**Fig. 11:**
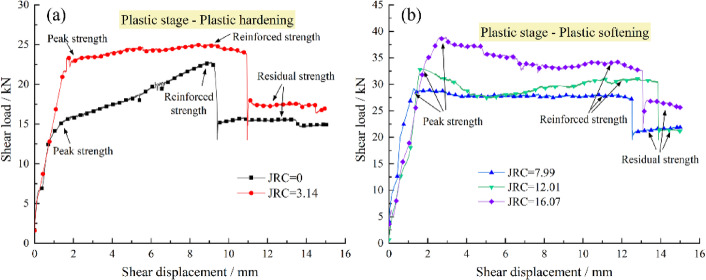
Behavioral characteristics in the plastic stage of bolted joints with different JRC. **a** Plastic hardening; **b** Plastic softening.

Plastic hardening, typically characterized by a recovery of strength that exceeds the initial peak value. This behavior, as illustrated in Fig. 11(a), typically occurs in joints with low to moderate JRC under low normal stress. Under these conditions, the joint itself exhibits a small post-peak stress drop or a ductile transition to residual strength. Concurrently, the pronounced dilatancy allowed by the low confining stress induces significant axial deformation in the bolt, enabling it to rapidly mobilize its shear and axial capacity. Crucially, the bolt’s increasing resistance not only compensates for the minor strength loss of the joint but also surpasses the initial peak strength of the system. This results in a net increase in the overall shear load, manifesting as the “plastic hardening” response where the reinforced strength exceeds the initial peak strength.

Plastic softening, typically characterized by a post-peak strength drop followed by a partial recovery. This behavior, as shown in Fig. 11(b), is characteristic of high-JRC joints under high normal stress. Here, the joint undergoes a large, brittle post-peak stress drop due to the rapid shearing off of asperities. While high JRC promotes dilatancy and bolt mobilization, the high normal stress constrains this dilation and the bolt’s capacity is mobilized at a finite rate. Consequently, the abrupt and substantial strength loss of the joint immediately after the peak cannot be instantaneously compensated by the bolt. This leads to an initial drop in the overall shear load. As shearing continues, the bolt enters its hardening phase and its resistance increases, pulling the system strength back up to a reinforced level. However, because the joint’s peak strength was so high and its degradation so severe, this reinforced strength typically remains below the original peak. The net effect is an overall “plastic softening” trend, characterized by a post-peak drop followed by a limited recovery.

It is noteworthy that normal stress significantly governs the shear mechanical behavior of bolted joints during the plastic stage by modulating joint dilatancy, which directly controls the mobilization of the bolt’s shear contribution. Under low confining stress, pronounced dilatancy enables the bolt to fully mobilize its capacity, resulting in a reinforced strength that surpasses the joint’s initial peak and produces a plastic hardening response. Conversely, high normal stress suppresses dilatancy and exacerbates the joint’s post-peak strength loss, which outpaces the bolt’s ability to compensate, thereby leading to plastic softening.

In essence, the transition from hardening to softening is governed by the ratio of the bolt’s reinforcement capacity to the joint’s post-peak strength degradation. Plastic hardening dominates when the bolt’s capacity is sufficient to over-compensate for the joint’s strength loss, whereas plastic softening occurs when the joint’s degradation overwhelms the bolt’s ability to compensate fully.

(3) Failure stage. In the failure stage, the shear load decreases nonlinearly and then drops abruptly due to bolt fracture. This phase corresponds to the Deformation - fracture stage on the bolt’s axial force curve. As the bolt cross-section contracts, its shear capacity declines, and the total shear load decreases rapidly after reaching the reinforced strength. The sudden load drop, typically between 6 kN to 10 kN, represents the total shear contribution of the bolt and depends on bolt deformation, joint morphology, normal stress, and experimental precision.

(4) Residual stage. Once the bolt fractures, the joint enters the residual stage, which is characterized by a nearly flat shear load-displacement curve. At this point, the bolt has lost all load-bearing capacity, thus the residual shear strength equals that of an unbolted joint under the same conditions.

In summary, the four stage elucidate the complete shear process of bolted rock joints by integrating the evolution of shear load, joint dilatancy, and critically, the real-time bolt axial force. This integrated analysis clarifies the distinct mechanical roles of the joint and the bolt at each stage: the joint surface primarily governs the initial elastic response, while the bolt’s progressive mobilization, through a combination of axial and shear forces, dictates the post-peak plastic behavior (hardening or softening), leads to the failure stage upon its fracture, and ultimately leaves the joint at its inherent residual strength.

The four stages described above represent the complete shear process of bolted rock joints. This four-stage model proposed in this study offers a distinct perspective compared to previous stage-division models for bolted joints^23^. While existing models typically categorize stages based on the macroscopic shear load-displacement curve of the joint system, our model is fundamentally rooted in the directly measured axial force evolution of the bolt. This bolt-centric approach allows us to identify a distinct initial adjustment-relaxation stage and to provide a mechanistic explanation for each stage based on the bolt’s material behavior and its interaction with the joint. Consequently, this model not only describes the macroscopic phenomena but also elucidates the underlying driver, namely the state of the bolt, which governs the progression of the shear process.

It is noteworthy that this study characterized joint morphology using the averaged three-dimensional JRC as a scalar roughness index. While this approach effectively captured the overall influence of roughness on dilatancy and bolt mobilization, it does not account for the anisotropy and specific morphological features described by true 3D roughness parameters^40^. Such parameters can influence the direction-dependent shear strength and the localized stress concentrations on the bolt. Future research incorporating a detailed analysis of 3D surface topography will be valuable to decouple the effects of overall roughness from asperity angularity and directionality on the bolt-rock interaction mechanism.

## Conclusions

This study systematically investigated the shear mechanical behavior of bolted joints and the rock-bolt mechanical interaction mechanism through direct shear tests under various conditions combined with full-process monitoring of the bolt axial force. The main conclusions are as follows:

The joint roughness coefficient (JRC) is a key factor influencing the shear behavior of bolted joints. A higher JRC induces a more pronounced dilatancy effect, which provides greater axial deformation space for the bolt, thereby more fully mobilizing its axial shear capacity. Consequently, both the peak strength and the reinforced strength of the bolted joint are significantly enhanced. However, an excessively high JRC can lead to premature bolt failure due to excessive deformation, ultimately reducing its ultimate shear displacement. Increased normal stress suppresses joint dilatancy, constrains the axial deformation space of the bolt, and thus diminishes the shear resistance mobilized by the bolt at identical shear displacements. The bolt inclination angle influences the normal displacement of the joint surface via a “guiding effect” and governs the distribution between the bolt’s axial force and shear force. Under the experimental conditions herein, a 45° inclination angle demonstrated the optimal performance by facilitating rapid mobilization of axial force while effectively engaging shear resistance.

Utilizing the axial force monitoring system to track the variation of bolt axial force throughout the shearing process, the mechanical response of the bolt can be divided into four characteristic stages based on the axial force-displacement curve: initial adjustment-relaxation, elastoplastic, hardening, and deformation-failure. This delineation reveals the deformation coordination mechanism and load evolution law of the bolt during shearing. Integrating the variations in shear load, dilatancy, and axial force, the overall shear process of the bolted joint is categorized into four stages: elastic, plastic, failure, and residual. The respective mechanical contributions of the joint surface and the bolt in each stage are clarified: In the elastic stage, shear resistance is primarily provided by the joint surface, with the bolt contributing mainly through its transverse (shear) force. In the plastic stage, the strength of the joint surface softens, and the bolt provides the primary shear contribution through the combined action of axial and shear forces, determining whether the stage exhibits a “plastic hardening” or “plastic softening” trend. The failure stage corresponds to the necking and fracture of the bolt. The residual stage exhibits a strength essentially identical to that of the unbolted joint.

By enabling real-time axial force monitoring, this research clarifies the deformation coordination and mechanical interaction mechanism between the bolt and the joint throughout the entire shearing process. The findings provide valuable insights for deepening the theory of rock bolting and improving support design practices.

## Data Availability

The data that support the findings of this study are available on request from the corresponding author.
